# Process-Outcome-Relationships and Solution-Based Interventions With Grandparent Caregivers

**DOI:** 10.1093/geroni/igaa057.1650

**Published:** 2020-12-16

**Authors:** Julian Montoro Rodriguez, Bert Hayslip, Jennifer Ramsey, Jane Jooste

**Affiliations:** 1 University of North Carolina at Charlotte, Charlotte, North Carolina, United States; 2 University of North Texas, Denton, Texas, United States; 3 UNC Charlotte, Charlotte, North Carolina, United States; 4 Lewisville School District, Lewisville, Texas, United States

## Abstract

Grandparents raising grandchildren face many challenges such as isolation, stigma, negative thoughts, and biases from age peers and service providers. In contrast, recent work suggests a need to re-frame grandparent caregiving in positive terms. In this light, the present study explored the processes key to the success of a solution-focused intervention with such persons. Fifty-two grandparents caregivers participated in a six-session solution focused intervention, using the theoretical framework of Selection, Optimization and Compensation (SOC) by Baltes & Baltes (1990). Throughout the program the emphases were on goal setting, solution focused thinking and communication skills. Participants were randomly assigned to intervention or waiting list control conditions. Within each session measures of hopefulness, positive thoughts about one’s grandchild, solution focused thinking, self-rated stress and perceived goal attainment were taken. Results indicated significant decreases in stress and increases over six sessions in hopefulness, positive thoughts about the grandchild, being solution-oriented and success in overcoming barriers interfering with goal setting and implementation (p<.05). Measures of goal accomplishment, strategy usage, success and satisfaction with effort were uniformly high. Such findings speak to key processes underlying the efficacy of a solution-focused intervention with grandparent caregivers. Measures of such processes also predicted outcome measures, e.g., resilience, positive affect, caregiver strain, well-being, loneliness, depression, parental efficacy and adequacy of service needs met. These findings reinforce the importance of understanding change processes as predictors of solution-focused intervention outcomes with grandparent caregivers and are consistent with the need to emphasize such persons’ strengths.

